# Astrocytic MicroRNA in Ageing, Inflammation, and Neurodegenerative Disease

**DOI:** 10.3389/fphys.2021.826697

**Published:** 2022-02-10

**Authors:** Aimee J. Chu, Joanna M. Williams

**Affiliations:** Department of Anatomy, Brain Health Research Centre, University of Otago, Dunedin, New Zealand

**Keywords:** astrocytes, microRNA, Alzheimer’s disease, Parkinson’s disease, amyotrophic lateral sclerosis, ageing, inflammation, neurodegeneration

## Abstract

Astrocytes actively regulate numerous cell types both within and outside of the central nervous system in health and disease. Indeed, astrocyte morphology, gene expression and function, alongside the content of astrocyte-derived extracellular vesicles (ADEVs), is significantly altered by ageing, inflammatory processes and in neurodegenerative diseases, such as Alzheimer’s disease, Parkinson’s disease and amyotrophic lateral sclerosis. Here, we review the relevant emerging literature focussed on perturbation in expression of microRNA (miRNA), small non-coding RNAs that potently regulate gene expression. Synthesis of this literature shows that ageing-related processes, neurodegenerative disease-associated mutations or peptides and cytokines induce dysregulated expression of miRNA in astrocytes and in some cases can lead to selective incorporation of miRNA into ADEVs. Analysis of the miRNA targets shows that the resulting downstream consequences of alterations to levels of miRNA include release of cytokines, chronic activation of the immune response, increased apoptosis, and compromised cellular functioning of both astrocytes and ADEV-ingesting cells. We conclude that perturbation of these functions likely exacerbates mechanisms leading to neuropathology and ultimately contributes to the cognitive or motor symptoms of neurodegenerative diseases. This field requires comprehensive miRNA expression profiling of both astrocytes and ADEVs to fully understand the effect of perturbed astrocytic miRNA expression in ageing and neurodegenerative disease.

## Introduction

Astrocytes are highly ramified glial cells found throughout the central nervous system (CNS). Originally thought to provide little more than structural support for neurons, it is now evident that astrocytes actively regulate numerous functions and cell types in both the healthy and diseased brain. Astrocytes are a fundamental component of both the tripartite synapse and blood-brain barrier (BBB). The intimate proximity of astrocytes to neurons and capillary wall-forming endothelial cells allows astrocytes to modulate and optimise the synaptic and wider CNS environment for current and future activity. Astrocytes regulate blood flow, distribute nutrients to nearby neurons, take up and recycle excess neurotransmitters and spent metabolites and release gliotransmitters, synaptogenic and neurotrophic factors and neurotransmitter precursors (Sofroniew and Vinters, 2010; Baldwin and Eroglu, 2017). Furthermore, significant crosstalk between astrocytes, microglia, other CNS cell types and CNS-non-resident cells is mediated by a variety of signalling molecules including growth factors and cytokines. This enables astrocytes, especially in response to CNS injury, to actively regulate the cellular responses and transcriptional profiles of other cell types while being regulated themselves in a similar manner (Linnerbauer et al., 2020; Matejuk and Ransohoff, 2020).

Intercellular communication is also facilitated by the release of astrocyte-derived extracellular vesicles (ADEVs). ADEVs, including exosomes and microvesicles, are vesicles of 30–1,000 nm containing proteins and functional genetic material (Valadi et al., 2007) encased by a lipid bilayer (Zhao et al., 2021). ADEVs can be internalised by neurons (Ibáñez et al., 2019; Xu et al., 2019), microglia (Neckles et al., 2019; Liao et al., 2020), oligodendrocytes (Willis et al., 2020), endothelial cells (Kriaučiūnaitė et al., 2021), and other astrocytes (Ipas et al., 2015), while ADEV transfer across the BBB enables astrocytes to influence cells beyond the CNS (Cai et al., 2017; Dickens et al., 2017). Differences between the cargo of ADEVs and the originating astrocytes (Ipas et al., 2015; Jovičić and Gitler, 2017) point to the existence of preferential packaging mechanisms (Groot and Lee, 2020), suggesting that curation of ADEV content is an important mechanism by which astrocytes communicate with other cells.

Following acute and chronic CNS injury, astrocytes undergo morphological, molecular and functional changes that are collectively termed reactive astrogliosis (Escartin et al., 2021). Whether these changes, such as the formation of a glial scar, are beneficial or detrimental depends on the severity and nature of the insult (Sofroniew and Vinters, 2010). Loss of normal astrocyte functions alongside gain of toxic functions can further exacerbate pathological processes (Sofroniew and Vinters, 2010). Neurodegenerative diseases, including Alzheimer’s disease (AD), Parkinson’s disease (PD) and amyotrophic lateral sclerosis (ALS), are characterised by CNS inflammation and deteriorating cognitive and/or motor function. These symptoms arise from the steady and irreversible degeneration of neurons in the brain or spinal cord, but the astrocytic contribution to the pathological processes underlying these chronic conditions is becoming increasingly recognised (Pekny et al., 2016). Dysregulated gene expression is a well-established feature of neurodegenerative disorders and normal ageing (Cooper-Knock et al., 2012). Recent work has highlighted the contribution of microRNA (miRNA), short non-coding RNAs that fine-tune gene expression by inhibiting protein translation, to neurodegenerative and ageing processes (Danka Mohammed et al., 2017; Shaik et al., 2018; Gagliardi et al., 2019; Goh et al., 2019). This includes a nascent literature evaluating the role of astrocyte-derived miRNA.

Here, we review accumulating evidence implicating disrupted miRNA expression in astrocytes and ADEVs in the pathophysiology of neurodegenerative diseases, highlighting downstream targets of the perturbed miRNA and the biological pathways affected. We build on recent reviews (Neal and Richardson, 2018; Bai et al., 2021) by considering alterations to astrocytic miRNA expression in ADEVs and that arising from ageing and inflammatory processes. It is important to note that studies utilising astrocytoma or glioblastoma cells to represent astrocytes were not included due to the well-documented differences in miRNA expression between primary astrocytes and cultures derived from astrocytoma cell lines (Lee et al., 2013; Zhou et al., 2017).

## MicroRNA Function and Regulation

Gene silencing is initiated following the interaction of a seed sequence (nucleotides 2–8) at the 5′ end of a mature miRNA with a perfectly complementary sequence in the 3′-untranslated region of a target mRNA (Chipman and Pasquinelli, 2019). The subsequent displacement of translation initiation complexes from the mRNA or recruitment of mRNA-degrading protein complexes ultimately results in reduced expression of the target protein (Iwakawa and Tomari, 2015). As most mammalian miRNAs are only partially complementary to their targets beyond the seed sequence, one miRNA can regulate numerous compatible mRNAs. Furthermore, one mRNA can be targeted by multiple miRNAs. Fascinatingly, both mature miRNA and precursor miRNA have been identified within EVs (Chen et al., 2010). Thus, perturbed miRNA expression is likely to have numerous downstream consequences.

Complex biogenesis paired with numerous transcriptional and post-transcriptional regulatory mechanisms provides multiple opportunities to regulate miRNA expression. Access of RNA polymerase II or transcriptional regulators to the genes encoding miRNA can be facilitated or restricted by epigenetic modifications, such as DNA methylation or chromatin remodelling and the binding of transcription factors (TFs) to miRNA promotor regions (Morales et al., 2017), leading to altered amounts of miRNA transcripts within cells. Notably, all of these mechanisms have been associated with various neurodegenerative conditions (Kwon et al., 2016; Berson et al., 2018). Furthermore, miRNA can regulate their own expression *via* positive and negative feedback loops (Zuo et al., 2016; Inukai et al., 2018; Ferro et al., 2019).

## Dysregulation of MicroRNA and Their Target mRNA in Astrocytes and Astrocyte-Derived Extracellular Vesicles in Neurodegenerative Diseases

Increasing evidence suggests that dysregulation of astrocytic and ADEV-associated miRNA is a common feature of neurodegenerative disease. Table 1 summarises studies showing a direct link between ageing, inflammation, neurodegenerative diseases or disease-relevant stimuli and altered expression of miRNA within astrocytes or ADEVs. Target genes are noted where investigated.

**TABLE 1:** Differentially expressed microRNA in astrocytes and astrocyte-derived extracellular vesicles in ageing, inflammation, and neurodegenerative disease.

Disease/condition	miRNA	Direction of change in treated or diseased astrocytes or ADEVs (*cf.* control)		Type of study	Target genes	Treatment/model	Study
		Astrocytes	ADEVs				

AD	miR-146a-(5p)	↑		Targeted: Northern blot	*IRAK1* ^b,d^	5 μM Aβ_42_ + 10 ng/mL IL-1β-treated HAG cells	Cui et al., 2010
AD	miR-155	↑		Targeted: RT-qPCR	*Socs1* ^b^	Aβ_42_ fibril (30 μM, 24 h) treated primary murine astrocytes	Guedes et al., 2014
AD	miR-146a	↑		Targeted: Northern blot and RT-qPCR	*CFH*^b,d^, *IRAK1*^b,d^, *TSPAN12*^b,d^	5 μM Aβ_42_ + 10 nM TNFα (1 week) treated HAG cells	Li et al., 2011
Ageing	**miR-16-(5p)**, **miR-17-(5p)**, **miR-140-(3p)**	↑		Exploratory: MMChIP assay with RT-qPCR validation	*Mapk3^b^*, *Ngfr^b^* (miR-206-3p), *Tnf-*α*^b^* (miR-181a-5p), *Sirt1^b^*, *Slc17a7^b^* (miR-138-5p)	HO-1 overexpression in primary rodent astrocytes	Lin et al., 2015
						
	**miR-29c-(3p)**, **miR-138-(5p)**, **miR-181a-(5p)**, **miR-187-(3p)**, **miR-206-(3p)**, **miR-297**	↓					
Ageing	miR-335-3p	↑		Targeted: RT-qPCR	*Hmgcs1*^a,d^, *Sfrs2*^a,d^	Young (7 DIV) cf. aged (35 DIV) primary murine astrocytes	Raihan et al., 2018
ALS	miR-21-(5p), miR-146a-(5p)	↓		Targeted: RT-qPCR	*Irak1*^b^, *Traf6*^b^ (miR-146a-5p)	Primary cortical astrocytes from mSOD1 mice	Gomes et al., 2019
ALS	miR-21-5p, miR-146a-5p, miR-155-5p	↓	↓	Targeted: RT-qPCR	No targets investigated	Primary cortical astrocytes from mSOD1 mice and secreted ADEVs	Gomes et al., 2020
						
		↑	↓			Primary spinal cord astrocytes from mSOD1 mice and secreted ADEVs	
ALS	TaqMan^TM^ Array Rodent microRNA A + B Cards v3.0	Not assessed	No change observed in any of the 752 miRNA investigated	Exploratory: TLDA RT-qPCR	No targets investigated	ADEVs from primary mSOD1 murine astrocytes	Jovičić and Gitler, 2017
ALS	**miR-494-3p**	Not assessed	↓	Exploratory: GeneChip array with RT-qPCR validation	*SEMA3A^b^*	ADEVs from patient-derived iAstrocytes (*C9orf72* mutation)	Varcianna et al., 2019
Inflammation	**let-7f-(5p)**, **miR-16-5p**, **miR-100-(5p)**, **miR-125a-5p**, **miR-125b-5p**	No change	↑	Exploratory: nCounter with RT-qPCR validation	*Ntrk3*^a,d^, *Bcl2*^a,d^ (miR-125a-5p and miR-16-5p)	IL-1β (200 ng/mL, 2 h) treated primary rodent astrocytes and secreted ADEVs	Chaudhuri et al., 2018
Inflammation	**miR-16-5p**, **miR-107**, **miR-125a-5p**, **miR-125b-5p**, **miR-145-(5p)**	No change	↑	Exploratory: nCounter with RT-qPCR validation	*Ntrk3*^a,d^, *Bcl2*^a,d^ (miR-125a-5p and miR-16-5p)	TNFα (200 ng/mL, 2 h) treated primary rodent astrocytes and secreted ADEVs	Chaudhuri et al., 2018
Inflammation	**miR-30d-(5p)**, **miR-141-3p**	↑	↑	Exploratory: TLDA RT-qPCR with ddPCR validation	No targets investigated	IL-1β (10 ng/mL, 24 h) treated primary human astrocytes and secreted ADEVs	Gayen et al., 2020
Inflammation	miR-146a	↑		Targeted: RT-qPCR	*IRAK1*^b,d^, *TRAF6*^b^	IL-1β (10 ng/mL, 24 h) treated primary human astrocytes	Iyer et al., 2012
Inflammation	miR-23a, miR-146a, miR-155	↑		Exploratory: TLDA RT-qPCR with RT-qPCR validation	*CD47*^a^ (miR-155)	IL-1β (0.05 μg/mL, 24 h) or TNFα (0.01 μg/mL, 24 h) treated primary human astrocytes	Junker et al., 2009
Inflammation	miR-155-5p	↑		Targeted: RT-qPCR	No targets investigated	IL-1β (10 ng/mL, 24 h) treated primary human astrocytes	Korotkov et al., 2018
Inflammation	miR-146a-5p	↑		Targeted: RT-qPCR	*Traf6* ^a,d^	IL-1β or TNFα (both 10 ng/mL, 6–24 h) treated primary murine astrocytes	Lu et al., 2015
Inflammation	**miR-125b-(5p)**	↑		Exploratory: Fluorescent miRNA array panels with Northern blot validation	*CDKN2A* ^d^	IL-6 (10 μM, 18 and 36 h) treated normal human astrocytes	Pogue et al., 2010
Inflammation	**miR-155-(5p)**, miR-155* (miR-155-3p)	↑		Exploratory: Illumina microarray with RT-qPCR validation	*SOCS1*^b,d^ (miR-155-5p)	IL-1β or TNFα (both 10 ng/mL, 24 h) treated primary human astrocytes	Tarassishin et al., 2011
Inflammation	miR-21-(5p), miR-146a-(5p), miR-155-(5p)	↑		Targeted: RT-qPCR	No targets investigated	IL-1β (10 ng/mL, 24 h) treated primary human astrocytes	van Scheppingen et al., 2016
Inflammation	miR-146a, **miR-147b**	↑		Exploratory: RNA sequencing with RT-qPCR validation	No targets investigated	IL-1β (10 ng/mL, 24 h) treated primary human astrocytes	van Scheppingen et al., 2018
PD	miR-155-(5p)	↑		Targeted: RT-qPCR	*Socs1* ^b^	IFN-γ (10 ng/mL, 3 h) treated primary murine DJ-1 KO astrocytes	Kim et al., 2014
PD	miR-151-3p, miR-182-5p, miR-222-3p, miR-423-3p	Not assessed	↑	Exploratory: RNA sequencing with RT-qPCR validation	*Map2k4*^a,d^ (miR-200a-3p)	ADEVs from MPP+ (4 mM, 4 days) treated primary murine astrocytes	Shakespear et al., 2020
							
	miR-15a-5p, miR-23a-5p, miR-138-5p, miR-150-5p, miR-194-5p, **miR-200a-3p**, miR-1247-5p, miR-3966		↓				

*^a^Luciferase assay.*

*^b^RT-qPCR.*

*^c^Northern blot.*

*^d^Western blot.*

*Bold text denotes validated miRNA. Brackets denote additional miRNA details gathered from primer, probe, inhibitor, or miRNA sequences provided in the original articles. HAG, human astroglial cells; DIV, days in vitro; TLDA, TaqMan^TM^ Low Density Array; ddPCR, droplet digital PCR.*

### Dysregulation of MicroRNA in Astrocytes in Normal Ageing

Increasing age is an established risk factor for the development of most neurodegenerative conditions. Even normal ageing is often accompanied by reductions in cognitive function, underpinned by impaired synaptic communication, altered metabolism and mitochondrial dysfunction in ageing neurons (Hou et al., 2019; Temido-Ferreira et al., 2019). Furthermore, the mild inflammatory state of the ageing brain (Palmer and Ousman, 2018) is likely driven by non-neuronal cells. Notably, ageing is associated with morphological, molecular and functional changes in astrocytes (Palmer and Ousman, 2018; Willis et al., 2020). Furthermore, RNA-sequencing of astrocytes from aged wildtype mice reveals that ageing induces significant changes in gene expression (Boisvert et al., 2018; Habib et al., 2020). However, little is known about ageing-induced alterations to miRNA expression in astrocytes. Nevertheless, two *in vitro* studies have reported dysregulated astrocytic miRNA expression in response to models of non-pathological ageing.

**FIGURE 1:**
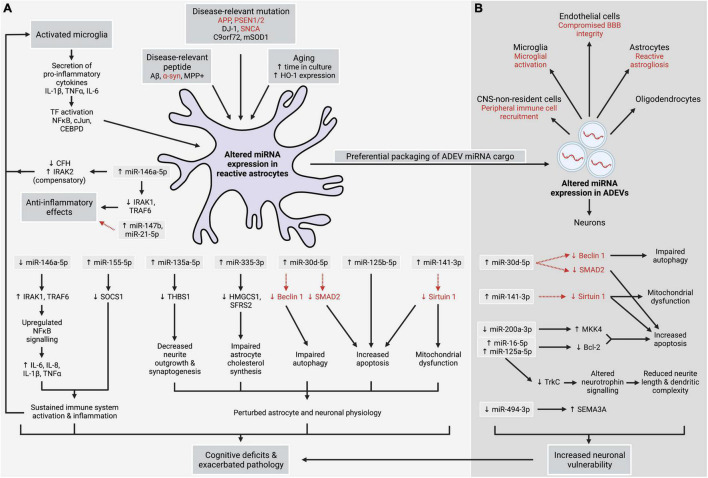
Altered expression of miRNA in astrocytes **(A)** and astrocyte-derived extracellular vesicles (ADEVs) **(B)**, arising from neurodegenerative, inflammatory, and ageing processes, contributes to sustained central nervous system (CNS) inflammation, neuronal injury, dysregulated autophagy, and mitochondrial dysfunction in astrocytes and neurons, and increased apoptosis. Uptake of ADEVs by neurons and other cell types is suggested to have numerous downstream effects. Together, these insults can exacerbate CNS pathology and contribute to the cognitive and motor symptoms observed in neurodegenerative conditions, indicating that dysregulated expression of astrocytic miRNA plays a central role in neurodegenerative disease. Alternatively, anti-inflammatory effects are observed in response to certain stimuli. Red font and dashed red arrows denote postulated links.

One study modelling normal ageing by extended culture (35 days) of primary murine astrocytes observed significant elevation of miR-335-3p compared to younger (7 days) astrocytes (Raihan et al., 2018). A concomitant decrease in the expression of direct targets HMGCS1 and SFRS2 was also observed, alongside reduced astrocytic cholesterol levels. Delivery of astrocyte-derived cholesterol to neurons *via* lipoproteins is essential for synaptic formation and function (Pfrieger, 2003), and HMGCS1 and SFRS2 are critical components of the cholesterol production pathway. Notably, impaired cholesterol synthesis in astrocytes arising from overexpression of miR-335-3p led to a downregulation of the essential synaptic protein PSD95 in neurons, while reducing miR-335-3p levels in the hippocampus of aged mice raised PSD95 protein levels and cholesterol production and was associated with improved performance on learning and memory tasks (Raihan et al., 2018; Figure 1A).

Heme oxygenase-1 (HO-1) is an inducible enzyme involved in the heme catabolism pathway. HO-1 expression is positively correlated with increasing age in post mortem brain with no observable neuropathology (Hirose et al., 2003). Astrocytes overexpressing HO-1 had significantly higher levels of three miRNA, while six miRNA were downregulated (Lin et al., 2015; Table 1). Tumour necrosis factor-alpha (*Tnf-*α), nerve growth factor receptor (*Ngfr*), mitogen-activated protein kinase 3 (*Mapk3*), Sirtuin 1 (*Sirt1*), and *Slc17a7*, which encodes the glutamate transporter VGLUT1, are known targets of the downregulated miRNA and were significantly upregulated in HO-1-overexpressing astrocytes (Lin et al., 2015). This suggests that multiple processes related to both astrocytic and neuronal physiology may be dysregulated as a result of age-related increases in HO-1 expression and accompanying miRNA dysregulation. Interestingly, upregulation of HO-1 expression, predominantly in astrocytes, has also been observed in neurodegenerative conditions, such as AD and PD after accounting for age and post mortem delay (Schipper, 2000). Thus, HO-1-induced alterations to astrocytic miRNA expression may contribute to the increased risk of developing a neurodegenerative disease in older age.

### Dysregulation of MicroRNA in Astrocytes in Response to Inflammation

Considerable evidence demonstrates that miRNA expression is altered in astrocytes and/or ADEVs following exposure to the pro-inflammatory cytokines interleukin-1-beta (IL-1β), tumour necrosis factor-alpha (TNFα) and interleukin-6 (IL-6) (Junker et al., 2009; Pogue et al., 2010; Tarassishin et al., 2011; Iyer et al., 2012; Lu et al., 2015; van Scheppingen et al., 2016, 2018; Chaudhuri et al., 2018; Korotkov et al., 2018; Gayen et al., 2020; Table 1). Release of IL-1β, TNFα and IL-6 by activated microglia has been observed in AD and PD, while reactive microglia and elevated levels of these and other cytokines have been observed in ALS (Smith et al., 2012). Furthermore, activated astrocytes also release these cytokines (Choi et al., 2014; van Scheppingen et al., 2018), contributing to the chronic inflammatory state observed in neurodegenerative diseases.

Exposure of astrocytes to IL-1β and TNFα upregulates the expression of numerous miRNA (Table 1), including miR-146a-5p and miR-155-5p. Interestingly, co-upregulation of miR-146a-5p and miR-155-5p has been observed for both IL-1β- and TNFα-treated astrocytes (Junker et al., 2009; van Scheppingen et al., 2016; Table 1), in line with observations that these miRNA can act in concert to refine cellular responses to inflammation (Mahesh and Biswas, 2019). Nuclear factor kappa-light-chain-enhancer of activated B cells (NFκB) is an inflammation-associated TF that stimulates transcription of pro-inflammatory cytokines, including IL-6, IL-8, IL-1β, and TNFα (Saba et al., 2014; Shi and Sun, 2018; Figure 1A). Induction of miR-146a-5p is dependent upon NFκB signalling (Taganov et al., 2006), however, a negative feedback loop allows miR-146a-5p to temper the immune response by directly targeting interleukin-1 receptor-associated kinase 1 (IRAK1) and tumour necrosis factor receptor-associated factor 6 (TRAF6) which upregulate NFκB activity (Figure 1A). In contrast, miR-155-5p augments inflammation by reducing the expression of suppressor of cytokine signalling 1 (SOCS1), a protein that inhibits inflammatory signalling and the production of pro-inflammatory mediators (Cardoso et al., 2012; Figure 1A).

IL-1β also upregulates miR-147b and miR-21-5p expression in astrocytes (van Scheppingen et al., 2018; Table 1). Increased expression of miR-147b downregulates production of IL-6 and other pro-inflammatory cytokines in astrocytes and macrophages (Liu et al., 2009; van Scheppingen et al., 2018) suggesting that miR-147b is a negative regulator of inflammation. On the other hand, while miR-21-5p can both silence and promote NFκB signalling (Ma et al., 2011), it is generally reported to have anti-inflammatory properties in the CNS (Gaudet et al., 2018), and has been shown to reduce the hypertrophic response of astrocytes to spinal cord injury (Bhalala et al., 2012; Figure 1A).

IL-1β or TNFα also alter the miRNA cargo of ADEVs and stimulate ADEV production (Dickens et al., 2017; Chaudhuri et al., 2018; Gayen et al., 2020). Significant upregulation of 27 miRNA was observed in ADEVs following treatment of astrocytes with IL-1β, with bioinformatics analysis of miRNA targets highlighting cell death and survival as a key pathway likely affected by these changes (Gayen et al., 2020). Further investigation confirmed the IL-1β-induced upregulation of miR-30d-5p and miR-141-3p in both ADEVs and astrocytes (Gayen et al., 2020; Table 1 and Figures 1A,B). Augmented miR-30d-5p has been shown to upregulate apoptosis and downregulate autophagy by respectively targeting SMAD2 (Yu and Liu, 2020) and the autophagosome-associated protein Beclin 1 (Zhao et al., 2017), while increased miR-141-3p, which downregulates the neuroprotective protein Sirtuin 1, was associated with increased apoptosis and mitochondrial dysfunction (Zheng et al., 2020). Thus, similar effects could occur in both astrocytes and ADEV-ingesting cells (Figures 1A,B). In another study, ADEVs enriched in miR-16-5p, miR-125a-5p, and miR-125b-5p secreted by IL-1β- and TNFα-treated astrocytes reduced neurite length and dendritic complexity in primary neurons (Chaudhuri et al., 2018). Both miR-16-5p and miR-125a-5p target two key components of the neurotrophin signalling pathway—*Ntrk3*, which encodes the neurotrophin TrkC receptor, and anti-apoptotic protein Bcl-2 (Figure 1B). Inhibiting miR-16-5p and miR-125a-5p in ADEVs abolished synaptic injury and restored neuronal activity. In contrast to Gayen et al. (2020), no changes to cellular miRNA expression in IL-1β- or TNFα-treated astrocytes were observed in this study (Chaudhuri et al., 2018). This, alongside discrepancies in the specific miRNA altered by IL-1β treatment between these studies, may reflect differences between species or in the duration and concentration of IL-1β used (Table 1).

Finally, IL-6 exposure was found to induce both astrogliosis and a significant increase in astrocytic miR-125b-5p (Pogue et al., 2010; Table 1), a miRNA that has been associated with apoptosis, inflammation and oxidative stress that is upregulated in the AD brain (Jin et al., 2018; Figure 1A).

### Dysregulation of MicroRNA in Astrocytes in Alzheimer’s Disease

Amyloid-beta (Aβ) accumulates in the AD brain due to impaired clearance mechanisms (Mawuenyega et al., 2010; Wildsmith et al., 2013), with monomers aggregating to form synaptotoxic oligomers and protofibrils (Klyubin et al., 2012) and ultimately forming the extracellular deposits of aggregated Aβ that are a characteristic hallmark of AD. Astrocytes have been shown to surround these plaques (Olabarria et al., 2010; Perez-Nievas and Serrano-Pozo, 2018) and ingest Aβ molecules (Jones et al., 2013; Söllvander et al., 2016). Exposure to aggregated Aβ induces reactive astrogliosis and increases astrocytic production of inflammatory mediators (Singh et al., 2020), while ingestion of aggregated Aβ can impair lysosome function, hinder astrocytic degradation of Aβ protofibrils and alter the cargo of ADEVs (Söllvander et al., 2016).

Accumulating evidence shows that *in vitro* exposure of astrocytes to Aβ alters miRNA expression. For example, miR-155 was significantly elevated in astrocytes treated with Aβ fibrils (Guedes et al., 2014; presumably miR-155-5p). Aβ can activate c-Jun N-terminal kinase (JNK), which subsequently activates the TF c-Jun (Yarza et al., 2016). Indeed, the Aβ-mediated increase in miR-155-5p in astrocytes was reduced when c-Jun was silenced (Guedes et al., 2014). Furthermore, expression of both c-Jun and miR-155-5p was upregulated in the brains of 3xTg transgenic AD mice. Notably, Aβ pathology was associated with decreased expression of the miR-155-5p target *Socs1* (Figure 1A), likely contributing to an increase in inflammatory processes in the AD brain alongside increased expression of IL-6 (Guedes et al., 2014) and other pro-inflammatory molecules.

Additionally, significant elevation of miR-146a-5p was observed following exposure of human astrocytes to Aβ, either alone or in conjunction with IL-1β (Cui et al., 2010; Table 1) or TNFα (Li et al., 2011). This occurred alongside increased activation of NFκB (Cui et al., 2010). Increased miR-146a-5p was associated with decreased expression of miR-146a-5p targets complement factor H (CFH), TSPAN12 and IRAK1 (Li et al., 2011), while increased IRAK1 levels were observed following inhibition of miR-146a-5p (Cui et al., 2010). Interestingly, a compensatory increase in IRAK2, which also upregulates NFκB signalling (Saba et al., 2014; Shi and Sun, 2018), was observed in both AD brain and Aβ-treated astrocytes (Cui et al., 2010) and is likely to counter any protective effects of IRAK1 downregulation. Furthermore, as CFH acts as an inhibitor of the complement cascade, the miR-146a-5p-mediated downregulation of CFH combined with IRAK2 upregulation following astrocytic exposure to Aβ is likely to result in sustained activation of the immune response, with the enhanced astrocytic production and secretion of NFκB-regulated pro-inflammatory cytokines contributing to continued activation of both microglia and astrocytes (Li et al., 2011; Figure 1A).

Increased expression of the cytokine-responsive TF CCAAT-enhancer binding protein-delta (CEBPD) has been observed in the brains of AD patients (Li et al., 2004), the APP/PS1 AD mouse model (Ko et al., 2012) and, notably, in astrocytes treated with pro-inflammatory cytokines (Chu et al., 2016) or surrounding Aβ plaques (Li et al., 2004). CEBPD induces transcription of miR-135a-5p, a miRNA that negatively regulates thrombospondin 1 (THBS1), a neurotrophic factor secreted by astrocytes to enhance neurite outgrowth and synaptogenesis (Sofroniew and Vinters, 2010; Chu et al., 2016; Figure 1A). Increased miR-135a-5p expression has been reported in the brains of APP/PS1 mice (Chu et al., 2016). Reducing CEBPD levels or inhibiting miR-135a-5p in APP/PS1 mice improved performance on learning and memory tasks.

### Dysregulation of MicroRNA in Astrocytes in Parkinson’s Disease

Though PD is characterised by the progressive loss of dopaminergic neurons in the substantia nigra resulting in movement dysfunction, astrocytes are also affected by PD pathophysiology (Booth et al., 2017), including the ingestion of neurotoxic aggregates of alpha-synuclein (α-syn) protein (Wakabayashi et al., 2000; Rostami et al., 2017), a key component of the neuronal Lewy bodies present in PD.

Mutations in the α-syn-encoding gene *SNCA* are associated with increased risk of developing PD (Campêlo and Silva, 2017), while *DJ-1* mutations give rise to a rare inherited form of PD, likely due to loss of numerous protective functions of the DJ-1 protein (Kahle et al., 2009; Giaime et al., 2010; Ariga et al., 2013). Investigation into the effects of α-syn dysfunction, aggregation and *SNCA* mutations on astrocytic miRNA expression is surprisingly lacking. However, interferon gamma (IFN-γ)-treated astrocytes from DJ-1 knock out mice demonstrate a robust increase in miR-155-5p and concomitant downregulation of *Socs1* (Figure 1A), suggesting that *DJ-1* mutations in familial PD may alter astrocytic regulation of miR-155-5p levels in response to inflammation (Kim et al., 2014; Table 1). As mentioned, elevated miR-155-5p and reduced *Socs1* expression was observed following exposure of astrocytes to Aβ (Guedes et al., 2014), suggesting that miRNA-mediated disruption of normal astrocyte responses to CNS inflammation may be a key feature of both diseases.

The neurotoxin 1-methyl-4-phenyl-1,2,3,6-tetrahydropyridine (MPTP) and metabolite MPP+ are taken up into neurons *via* dopamine transporters, resulting in selective degeneration of dopaminergic neurons accompanied by parkinsonian symptoms (Kitayama et al., 1998) and increased expression of α-syn protein in differentiated PC12 cells (Zheng et al., 2020). Interestingly, exposure of astrocytes to MPP+ induces astrogliosis and the production of pro-inflammatory cytokines (Yu et al., 2016) and alters the miRNA cargo of ADEVs (Shakespear et al., 2020; Table 1). One notable change in ADEVs is the downregulation of miR-200a-3p, which targets the apoptosis-associated kinase *Map2k4*. Reduced transfer of miR-200a-3p to neurons *via* ADEVs rendered neurons more vulnerable to neurotoxic stimuli (Shakespear et al., 2020; Figure 1B). Similarly, exposing dopaminergic neurons to ADEVs enriched in miR-34a-5p increased neuronal susceptibility to neurotoxins due to the negative regulation of Bcl-2 by miR-34a (Mao et al., 2015; presumably miR-34a-5p). As miR-34a-5p expression was induced by exposing astrocytes to the inflammatory stimulus lipopolysaccharide, the presence of other ADEV components that may have contributed to neuronal loss cannot be ruled out. However, co-administration of miR-34a-5p inhibitors alongside miR-34a-5p-enriched ADEVs reduced neuronal vulnerability to neurotoxins (Mao et al., 2015). Interestingly, elevated expression of miR-34a-5p has also been observed in the brains of sporadic AD patients (Zhao et al., 2019) and in APP/PS1 mice (Wang et al., 2009).

### Dysregulation of MicroRNA in Astrocytes in Amyotrophic Lateral Sclerosis

ALS is characterised by progressive paralysis and muscle atrophy arising from the degeneration of motor neurons in the brain and spinal cord (Joilin et al., 2019). While the mechanisms underlying this degeneration are not completely elucidated, both astrocytes and ADEVs are known to play a significant role (Pehar et al., 2017; Chen et al., 2019). Although more than 20 genes are associated with ALS, mutations in the *SOD1* and *C9orf72* genes are among the most common and play a causative role in the development of both familial and sporadic forms of ALS (Tripolszki et al., 2017). Alongside this, cytosolic accumulation and aggregation of TAR DNA-binding protein 43 (TDP-43) is present in the majority of patients with familial or sporadic ALS. While TDP-43 is known to sequester miRNA in neurons (Zuo et al., 2021), there is no current literature exploring its role in the regulation of miRNA in astrocytes.

Altered expression of several miRNA has been observed, however, in both primary astrocytes and ADEVs from the SOD1-G93A (mSOD1) mouse model of ALS (Gomes et al., 2019, 2020), though, curiously, others found no differences in ADEV cargo between mSOD1 and wildtype mice (Jovičić and Gitler, 2017). Interestingly, Gomes et al. (2019, 2020) reported that the direction of change was dependent on the CNS region from which the astrocytes were derived. Cortical mSOD1 astrocytes had reduced levels of miR-21-5p and miR-146a-5p (Gomes et al., 2019) (Table 1), whereas increased expression of both these miRNA and miR-155-5p were identified in spinal cord mSOD1 astrocytes (Gomes et al., 2020). Markedly, all three miRNA were downregulated in mSOD1 ADEVs regardless of the region of origin (Gomes et al., 2020). Reduced expression of anti-inflammatory miRNAs miR-146a-5p and miR-21-5p in cortical mSOD1 astrocytes was proposed to contribute to the neuronal damage and mitochondrial dysfunction observed following co-culture of reactive astrocytes and motor neurons (Gomes et al., 2019).

Another study identified alterations to the miRNA cargo of ADEVs secreted by directly reprogrammed human astrocytes (iAstrocytes) derived from ALS patient fibroblasts with the *C9orf72* mutation compared to ADEVs from control iAstrocytes (Varcianna et al., 2019). One significantly downregulated miRNA was miR-494-3p, which targets *SEMA3A* mRNA (Figure 1B). Though SEMA3A acts as a guidance signal for developing axons (Carulli et al., 2021), aberrant SEMA3A expression has been observed in ALS (Körner et al., 2016) and is suggested to contribute to neuronal apoptosis (Carulli et al., 2021). Notably, uptake of ALS patient-derived ADEVs by motor neurons induced neuronal death (Varcianna et al., 2019), likely due to a reduction in miR-494-3p levels in neurons following ADEV internalisation. Accordingly, restoring neuronal levels of miR-494-3p with a mimic reduced *SEMA3A* expression and increased neurite length and motor neuron viability (Varcianna et al., 2019).

Interestingly, there were no miRNA alterations in common between astrocytes from the mSOD1 and *C9orf72* models, suggesting that the genetic basis of ALS leads to differential effects on ADEV miRNA cargo. Mutation-associated differences in ADEV secretion have also been observed. Fewer ADEVs were released by C9orf72 iAstrocytes (Varcianna et al., 2019), whereas primary murine mSOD1 astrocytes had enhanced ADEV secretion (Basso et al., 2013).

## Discussion

As summarised in Figure 1, significant alterations to astrocytic miRNA expression arise from the pathological processes underlying or contributing to neurodegenerative disease, including genetic mutations, accumulation of aggregated peptides, pro-inflammatory cytokine release by activated microglia, and ageing. Altered activity of key TFs in astrocytes appears to result in aberrant transcription of miRNA which ultimately affects the health and function of surrounding cells, particularly neurons. Furthermore, the curated nature of alterations to ADEV-associated miRNA indicates that ADEV-mediated intercellular communication is an important component of the astrocyte response to neurodegeneration with the potential for widespread, cell non-autonomous effects and propagation of pathology throughout the brain because of ADEV uptake. Many of the genes targeted by dysregulation of astrocytic and ADEV-associated miRNA converge upon common pathways including apoptosis, inflammation, TF signalling, and the loss of normal or neuroprotective astrocytic functions or proteins. This may underpin chronic CNS inflammation and increase neuronal susceptibility to neurodegenerative processes which may hasten disease onset and exacerbate symptoms. Furthermore, significant crosstalk between astrocytes and other cell types, as well as perturbed regulation of miRNA transcription, may amplify the effects of disrupted astrocytic miRNA expression.

Our understanding of perturbed astrocytic miRNA expression in neurodegenerative disease remains limited, especially in regard to ageing. Discrepancies regarding which miRNA are significantly altered in astrocytes in neurodegenerative disorders will hopefully be resolved with further investigation. Furthermore, while various alterations to the protein cargo of plasma ADEVs from AD patients have been reported (Goetzl et al., 2016, 2018; Winston et al., 2019), no studies have investigated miRNA changes in ADEVs in AD. There is also a profound lack of investigation into altered miRNA expression in astrocytes and ADEVs in relation to Huntington’s disease and non-Alzheimer’s type dementias, such as frontotemporal dementia and vascular dementia.

Research has largely focussed on small groups of differentially expressed miRNA and their mRNA targets, often selected based on known relationships to specific cellular processes or relevant target genes. Comprehensive miRNA expression profiling with methods such as RNA-sequencing in conjunction with bioinformatics tools would ensure that important changes in miRNA expression do not go undetected, while the use of up-to-date miRNA nomenclature will reduce ambiguity regarding which miRNA strand mediates the effects described. Researchers investigating disease-driven miRNA alterations in astrocytes should ensure the experimental model used is as relevant to the disease process as possible, ideally utilising human rather than rodent models and focussing on alterations caused by genetic mutations or causative agents pertinent to the disease of interest. Observations of ageing-induced alterations to miRNA expression highlight the critical importance of having age-matched healthy controls. Finally, while *in vitro* experiments assist in uncovering the mechanistic links between altered miRNA expression and downstream outcomes, the short time courses of such experiments should be married with longer term, *in vivo* observations from post mortem human brain or aged animal models to ensure the effects of dysregulated astrocytic miRNA expression are placed within the wider context of chronic neurodegenerative diseases.

## Author Contributions

AC and JW made substantial, direct, and intellectual contributions to the work, and approved it for publication.

## Conflict of Interest

The authors declare that the research was conducted in the absence of any commercial or financial relationships that could be construed as a potential conflict of interest.

## Publisher’s Note

All claims expressed in this article are solely those of the authors and do not necessarily represent those of their affiliated organizations, or those of the publisher, the editors and the reviewers. Any product that may be evaluated in this article, or claim that may be made by its manufacturer, is not guaranteed or endorsed by the publisher.
